# P021. Investigation on occipital headache associated with vertigo and vomiting discovers a familial clustering of Chiari I malformation and a “puzzle”

**DOI:** 10.1186/1129-2377-16-S1-A172

**Published:** 2015-09-28

**Authors:** Alessandro Panconesi, Marco Macucci, Maria L Bartolozzi, Stefania Mugnai, Leonello Guidi

**Affiliations:** Headache Center, Department of Neurology, Health Authority 11, Empoli, Italy

A 16-year-old male (patient 4) experienced an episode of bilateral parietal headache, preceded by vertigo and associated with vomiting, lasting about two weeks. An MRI scan performed for a subsequent episode of occipital and neck pain with vomiting and vertigo showed an imaging to the limit of Chiari I malformation (CMI): cerebellar tonsils below the foramen magnum, not reaching posterior arch of C1, but with obliteration of peribulbar posterior liquoral space, cyst septum pellucidum and cavum vergae. Left facial nerve palsy at the age of 12, frequent alimentary vomiting and abdominal pain, episodes of exercise-induced asthma although not confirmed through respiratory tests, were found in the patient's history.

An MRI was performed on his father for otoneurological symptoms characterized by a recurrent sensation of disequilibrium when suddenly changing position, on one occasion preceded by a sensation of right ear pressure (diagnosed as sudden hearing loss). Two years ago he experienced an episode of stabbing occipital headache with shoulder pain irradiation, nausea and phonophobia. The MRI revealed cerebellar tonsils slightly below the foramen magnum, to the limit of CMI. MRI was performed also on his mother (patient 1), who suffers from tension-type headache even for long periods of time (months), showing no alterations, and on his 21-year-old brother (patient 5) suffering from episodic tension-type headache, showing cerebellar tonsils slightly below the foramen magnum, to the limit of CMI.

**Figure 1:**
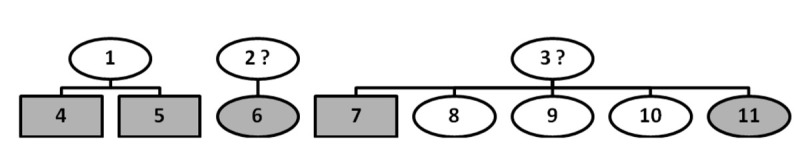
oval = female, rectangle = male, shaded = tonsillar descent diagnostic or to the limit of CMI, ? = MRI not performed

His mother's interview revealed that her sister (patient 3) has five sons, two of them with CMI: 1) a 13-year-old male (patient 7), suffering from West syndrome, who at 3 years of age had an MRI that showed approximately 10 mm caudal descent of cerebellar tonsils with a reduction of the liquoral flow at the craniocervical junction and associated syringomyelia extending from C6 to D2 vertebral body; 2) a 26-year-old female (patient 11) with a cerebellar tonsils extending 11 mm below the foramen magnum, associated with posterior fossa hypoplasia. The “puzzle” comes from the fact that both the husbands of the two sisters (patients 1, 3) have the same surname but with no recognized relationship. The doubt seems to be resolved by the MRI performed on the 34-year-old daughter (patient 6) of the third sister (patient 2) showing slight tonsillar descent but without obstacle to liquoral flow (Figure 1).

This report adds to other descriptions of familial clustering of CMI malformations, which suggest an underlying genetic basis.

Written informed consent to publish was obtained from the patient(s).

